# Overcoming Liability of Outsidership Under Sanctions: A Capability-Based Framework from Emerging Market Pharmaceuticals

**DOI:** 10.5812/ijpr-169173

**Published:** 2026-06-28

**Authors:** Sheida Paknejad, Mahdi Mohammadzadeh

**Affiliations:** 1 Department of Pharmacoeconomics and Pharma Management, School of Pharmacy, Shahid Beheshti University of Medical Sciences, Tehran, Iran

**Keywords:** Organizational Capabilities, Liability of Outsidership, Internationalization, Pharmaceutical Industry, Emerging Economies, Sanctions

## Abstract

**Background:**

Sanctions and economic constraints impose substantial barriers to firm internationalization in emerging economies, particularly by exacerbating the liability of outsidership (LOO), defined as the disadvantages associated with exclusion from critical business networks. Despite extensive research on barriers to internationalization, limited attention has been paid to how firms in sanctioned economies leverage organizational capabilities to mitigate LOO at the micro level.

**Objectives:**

Drawing on the resource-based view, dynamic capabilities theory, and the Uppsala internationalization model, this study examined how pharmaceutical companies navigate LOO under sanctions through the strategic deployment of organizational capabilities.

**Methods:**

A qualitative multiple-case study was conducted in 10 Iranian pharmaceutical companies operating internationally under sanctions. Data were collected through interviews with senior executives and analyzed using thematic analysis.

**Results:**

The findings indicated three main interrelated routes through which capabilities mitigate LOO: 1) networking capability (NC) increases absorptive capacity (AC) by establishing trust-based relationships and facilitating knowledge exchange; 2) AC enhances operational capability (OC) by aligning operations with international benchmarks; and 3) NC, AC, and OC jointly reduce LOO by enabling access to alternative networks, strengthening operational legitimacy, and generating a competitive advantage.

**Conclusions:**

This study contributes to the internationalization literature by demonstrating how sanctions intensify LOO and necessitate the reconfiguration of firm capabilities. The findings show that dynamic capabilities interact with operational capabilities to support internationalization in constrained environments.

## 1. Background

Global organizations must continuously manage crises that affect business performance, supply chain management, and market entry (1). These crises include economic crises, natural disasters, pandemics, and political conflicts, among others. All such crises threaten business stability and development, particularly for firms operating internationally (2). For pharmaceutical companies, international sanctions represent a major form of external pressure that can intensify LOO by blocking financial transactions, logistics, technology acquisition, and the supply of active pharmaceutical ingredients. These pressures create substantial disruptions and uncertainty in business operations (3, 4). Such external pressures further exacerbate LOO by cutting companies off from vital networks, knowledge sharing, and global partnerships (5). To manage these external pressures and LOO, firms must rely on their operational and dynamic capabilities (6). In this regard, the resource-based view (RBV) posits that firms can create lasting competitive advantages and reduce the effects of LOO through strategic capabilities (7).

## 2. Objectives

This study provides a framework for managing crises in international business. It had three objectives: 1) to clarify the distinct roles of different organizational capabilities in reducing LOO, 2) to examine the relationships among these capabilities, and 3) to provide practical guidance for managers pursuing internationalization in constrained settings.

## 3. Methods

### 3.1. Theoretical Background and Conceptual Development

#### 3.1.1. Organizational Capabilities in Internationalization

In this study, the RBV provides the foundational resource logic by explaining how valuable, rare, and inimitable capabilities function as strategic assets that enable firms to mitigate the effects of LOO and sustain competitive advantage under sanctions (8). These capabilities vary across firms and evolve over time as routine activities are developed and modified (9). Accordingly, considerable attention has been given to dynamic capabilities, which focus on change, and operational capabilities, which emphasize implementation efficiency (10). Specifically, dynamic capabilities, particularly networking competence and absorptive capacity, are described in the literature as strategic adjustment mechanisms that help companies reconfigure resources and respond to sanctions-related disruptions (11). Mitrega et al. (11) define networking capability as the activities and routines a company uses to initiate, develop, or terminate business relationships for its own benefit. This capability is especially important in emerging markets (12). Similarly, absorptive capacity enables a firm to identify, assimilate, and apply new knowledge effectively (13). In contrast, operational capabilities enable companies to perform daily tasks efficiently. These capabilities include product design, production management, marketing strategy, and supply chain coordination (14).

#### 3.1.2. Capability-Based Strategies and Liability of Outsidership

Firms encounter numerous obstacles during internationalization. One major obstacle is LOO, which describes the difficulties that arise when firms are not part of relevant business and social networks. Without insider status, companies struggle to access other actors, resources, and information. This isolation intensifies LOO by restricting access to vital resources and partnerships, particularly in regulated sectors such as pharmaceuticals.

Firms use several approaches to address LOO. One common approach is to strengthen networking capabilities (11). In emerging markets such as Vietnam, this enables firms to access essential resources through partnerships with suppliers and government agencies (15). Partnerships with local companies can also facilitate entry into informal networks (11). Another way to access resources is through organizational learning, also referred to as absorptive capacity. This can be observed in the example of Chinese firms hiring foreigners to acquire external expertise (16). Portuguese companies have reduced LOO by managing alliances themselves (17). However, the same approach has faced challenges in Russia because of political issues (18). Some organizations develop internal skills and emphasize self-sufficiency to reduce their dependence on other entities. Chinese firms have invested substantially in local research and development to respond to US trade restrictions. These studies show that the success, speed, and durability of any strategy depend on the specific context.

This study integrates three complementary perspectives: LOO provides the network-position logic, RBV explains the strategic value of capabilities, and dynamic capabilities theory clarifies how firms reconfigure these capabilities under sanctions-induced exclusion.

### 3.2. Research Context and Case Selection

This qualitative study examined how pharmaceutical companies in Iran use organizational capabilities to overcome barriers under sanctions. The study adopted an abductive multiple-case design, allowing existing theoretical perspectives, such as dynamic capabilities and LOO, to inform data interpretation while enabling processual relationships to emerge and be refined through iterative engagement with empirical evidence. In line with qualitative case research traditions, the study developed theoretically informed propositions rather than statistically testable hypotheses (19). Data were obtained from 10 Iranian pharmaceutical manufacturers. The findings are presented as a theoretical framework illustrating the patterns followed by each company during internationalization.

Crises tighten borders in international business, highlighting the importance of identifying effective patterns. The main selection criteria were Iranian pharmaceutical manufacturers with foreign market experience and active research programs (Table 1). Sampling was purposive and theoretical. It included firms that differed in size, product type, and entry mode to create strong contrasts, such as small biotechnology companies and large generic producers. This diversity supported replication logic, as patterns identified in one case could be examined in others until no new insights emerged. Cases were drawn from the Syndicate of Iranian Pharmaceutical Industries database. Selection began with a small number of firms and continued as new themes appeared, stopping after the 10th case when additional interviews yielded no substantively new themes. To demonstrate theoretical saturation, the number of newly generated first-order codes declined substantially after the eighth interview, and no substantively new themes emerged by the 10th interview.

**Table 1.** Profile of Case Study Companies ^a^

Company	Firm Size (Employees)	Product Focus	Internationalization Mode(s)
**A**	520	Anticancer and high-risk drugs	Import, export, contract manufacturing
**B**	120	High-risk drugs	Import, export
**C**	200	Generic pharmaceuticals	Import, export
**D**	500	Generic manufacturing	Import, export, joint venture
**E**	25	Biotechnology	Import, export
**F**	> 1,000	Injectable drugs	Import, export, technology transfer, FDA approval
**G**	150	Generic pharmaceuticals	Import, export, technology transfer, FDA approval
**H**	54	Generic manufacturing	Import, export, joint venture, contract manufacturing
**I**	650	Raw materials	Import, export
**J**	85	Vaccines and generic pharmaceuticals	Import, export

^a^ Companies are anonymized as A-J. All firms had active international operations and were selected through theoretical sampling to ensure variation in size, product focus, and internationalization modes.

Senior managers with at least 5 years of international experience were selected as interviewees (Table 2).

**Table 2.** Characteristics of Interview Participants ^a^

Company	Position	International Business Experience (Years)	Interview Duration (Minutes)
**A**	CEO	8	55
**B**	CEO	8	45
**C**	CEO	10	65
**D**	Export manager	7	110
**E**	Commercial manager	10	75
**F**	CEO	15	60
**G**	Export manager	20	40
**H**	CEO	18	60
**I**	CEO	12	40
**J**	Export manager	11	70
**Mean**	-	11.9	62.0

^a^ All participants held senior management positions with 7 to 20 years of international business experience (mean, 11.9 years). Interviews were conducted in person, audio-recorded with informed consent, and transcribed verbatim. Interview duration ranged from 40 to 110 minutes (mean, 62 minutes).

### 3.3. Data Collection Procedures

Initial company details were obtained from the Syndicate of Iranian Pharmaceutical Industries. Data were collected through semi-structured interviews. The interview guide used open-ended questions on topics such as international strategies, challenges, opportunities, and previous successes. This format allowed the researcher to probe more deeply and ask follow-up questions based on participants’ responses. The guide was first tested with two managers outside the sample to improve clarity and alignment with the study objectives. Sample questions included: “Can you describe how your firm has developed or leveraged external relationships to improve access to international markets or critical resources during periods of constraint?” and “What operational adjustments or organizational improvements has your firm implemented to strengthen its position and credibility in foreign markets?” Probes were used to elicit concrete examples and clarify unclear points.

One senior executive from each company, typically the chief executive officer, export manager, or commercial manager, participated in a face-to-face interview. Senior managers were selected as the most appropriate informants because they possess strategic oversight of internationalization efforts, including networking capabilities, external relationships, and capability deployment in response to firm activities, which are central to the study’s focus on firm-level responses.

Interview sessions lasted 40 to 110 minutes. Interviews stopped after the 10th company, when theoretical saturation was reached. With permission, all interviews were audio-recorded and transcribed verbatim. The names of people and firms were replaced with codes to protect privacy (Table 2). Participants were assured of anonymity and data security. No payments or rewards were provided to prevent bias.

### 3.4. Data Analysis

This study primarily relied on a prefigured coding strategy; however, an emergent coding approach was also applied to capture potential new factors arising from the data. Coding was conducted using MAXQDA 2020 software. Thematic analysis was then used to identify patterns in the data. The analysis followed a six-step process: 1) reading the transcripts several times to become familiar with the content, 2) open coding to identify initial ideas, 3) grouping codes into possible themes, 4) checking themes for consistency, 5) naming and defining each theme, and 6) writing the final report. Axial coding examined links between themes, while selective coding refined the central narrative.

The qualitative data were analyzed using MAXQDA, leading to the identification of a hierarchical structure of themes, dimensions, categories, subcategories, and initial codes. Table 3 summarizes the core structure of the findings. The examples presented in the table are illustrative and demonstrate the coding logic; they do not include all extracted codes generated during the analysis process.

**Table 3.** Thematic Structure of Capabilities Derived from Qualitative Analysis

Capabilities and Dimensions	Main Categories	Subcategories	Example Codes
**Absorptive capacity**			
Acquisition	External knowledge sourcing	Market scanning, partner interactions	Trade fairs, global databases
Assimilation	Knowledge interpretation	Market analysis, opportunity evaluation	Competitor analysis, reports
Transformation	Knowledge reconfiguration	Localization, capability development	Technology transfer
Exploitation	Knowledge application	Product development, process improvement	GMP compliance
**Networking capability**			
Initiation	Relationship formation	Partner identification, initial contact	Emails, intermediaries
Maintenance	Relationship development	Trust building, interaction continuity	Commitment, follow-up
Termination	Relationship restructuring	Partner replacement, network adjustment	Switching partners
**Operational capabilities**			
Efficiency	Operational performance	Cost efficiency, process optimization	Cost control, lead time
Flexibility	Adaptive capability	Product adaptation, strategic change	Packaging modification
Execution	Export implementation	Entry mode execution, logistics management	Direct export, intermediaries

Two researchers coded the data independently to improve validity. Cohen’s kappa was 0.85, indicating high agreement; differences were resolved through discussion. Trustworthiness was addressed using Lincoln and Guba’s standards. Credibility was supported through member checking, in which three interviewees reviewed summaries, and prolonged field engagement. Transferability was supported by detailed contextual descriptions. Dependability was based on a full audit trail of transcripts and codes. Confirmability was supported by reflexivity notes used to track researcher influence. Triangulation strengthened credibility by cross-checking interview data with company documents and earlier studies (20).

## 4. Results

### 4.1. Findings and Proposition Development

To meet the study objectives and identify the capabilities that drive internationalization in a restricted and unstable setting, key statements from interviewees are presented below and linked to their codes.

### 4.2. Networking Capability and Absorptive Capacity

Networking capability opens access to diverse knowledge, accelerates knowledge transfer, and creates learning opportunities (11). It drives innovation and strongly enhances absorptive capacity (21). The interviews supported this link. The managing director of Company F stated: “Working with large companies has helped us grow. It has raised our business knowledge and skills. We now have more staff who master business topics and speak English well. Our negotiation and communication skills have also improved. All this has strengthened our market position and expanded our international ties.”

Trust built through networks reduces cultural and institutional barriers and facilitates knowledge exchange. Once trust is established, firms can identify, absorb, and apply external knowledge more quickly and accurately. This directly strengthens AC (22). The commercial director of Company E explained: “Our ongoing, positive contact with a leading global pharmaceutical firm led to a new drug that would have taken years to develop on our own.”

These empirical findings indicate a positive relationship between NC and AC, whereby trust within networks reduces cultural and institutional barriers and facilitates knowledge exchange. Accordingly, we propose the following:

P1: In the internationalization of pharmaceutical companies, networking capability has a positive effect on absorptive capacity.

### 4.3. Absorptive Capacity and Operational Capability

Internationalization exposes firms to new resources and knowledge. However, firms can use these inputs only when they have strong absorptive capacity (23). High AC enables companies to adopt global technologies, standards, and management methods and apply them to daily operations (24).

The interviews supported this view. Interviewee A stated: “Learning from foreign markets has built up both specific and general knowledge inside the company. This has upgraded our production facilities and sped up our processes. We now launch new products with far more experience.”

The CEO of Company F added: “Earning GMP certificates from the European Union has raised our skills and know-how. As a result, production quality has clearly improved.”

These points are consistent with Emre Yildiz et al. (25), who found that absorbing foreign knowledge strengthens operational areas such as production and supply chains. Based on this evidence, we propose the following:

P2: In the internationalization of pharmaceutical companies, absorptive capacity has a positive effect on operational capability.

### 4.4. Networking Capability and Operational Capability

From the relational view and the RBV, firms gain competitive advantage through external ties and network resources. Strong interfirm links provide better access to resources, lower risk, greater flexibility, and shared learning, all of which enhance operational capability (26).

The CEO of Company G explained: “Since our ties with suppliers and partners have grown stronger, everything runs more smoothly. We get raw materials faster. Problems are fixed quickly, and better coordination has raised our productivity.”

Networking capability helps firms reconfigure resources and open channels for external knowledge (27). These points are consistent with the interview evidence on supply-chain gains. These findings suggest the following:

P3: In the internationalization of pharmaceutical companies, networking capability has a positive effect on operational capability.

### 4.5. Absorptive Capacity and Overcoming Liability of Outsidership

Absorptive capacity drives organizational learning and is a core tool for overcoming LOO. It is even more important when foreign markets are volatile, uncertain, complex, and ambiguous (28). Such conditions widen the information gap between outsiders and local players.

AC narrows this gap by drawing on knowledge from global databases, trade fairs, and competitors. Interviewee C stated: “We buy basic data from ITC, UNICEF, the World Bank, exhibition partners, and market research firms. We also track top competitors. All this information makes it easier to pick the right products and countries and to enter new markets.”

This is consistent with Li and Leme Fleury (28), who showed that AC can reduce network isolation and LOO by improving market knowledge. AC also helps firms absorb local insights, including customer preferences, regulations, and business customs, through contact with suppliers, buyers, and officials. This knowledge builds local ties and reduces isolation (24).

The CEO of Company F noted: “Technology transfer has boosted our international work. When partners see that we deal with reputable suppliers, trust grows on both sides. This has helped us get past the limits set by external pressures.”

We therefore propose the following:

P4: In the internationalization of pharmaceutical companies, absorptive capacity has a positive effect on overcoming LOO.

### 4.6. Networking Capability and Overcoming Liability of Outsidership

A main cause of LOO is a lack of familiarity with relevant networks (29). Therefore, building connections is an important first step. The type and location of those networks are highly important (18, 30). NC enables access to local and global networks through ties with key players, such as local agents, business partners, and small and medium-sized enterprises (SMEs). This reduces isolation (31).

In Iran’s pharmaceutical sector, local representatives often serve as entry points. The CEO of Company I stated: “In some countries, we work through local partners. We encourage them to build their own networks, and through those links we export to other firms.”

NC also creates new networks through informal channels and platforms such as trade fairs. The export director of Company J noted: “We attend exhibitions because they help us make new contacts. One partner leads to another, and the network keeps growing.”

Trust-based relationships reduce cultural and institutional barriers and strengthen a firm’s position abroad (22). The export manager of Company H explained: “Reconnecting with old contacts, even those outside pharmaceuticals, opened new doors. It sped up our entry into other markets. The trust we built also helped other Iranian producers join later.”

Sanctions worsen LOO by blocking global banking systems, including SWIFT, and disrupting supply chains (4). NC helps firms manage these constraints by creating alternative routes, such as offices in third countries or ties with regional SMEs (32, 33). Interviewee E stated: “Partners in third countries have helped us bypass political restrictions.” The commercial manager of Company A added: “Our local partners, mostly SMEs, want to grow with us. Their eagerness makes them ideal for distributing our products despite sanctions.”

These points align with Hagedoorn and Duysters (22) and Burt and Soda (34), who found that NC reduces network barriers through strong relationships. These findings suggest the following:

P5: In the internationalization of pharmaceutical companies, networking capability has a positive effect on overcoming LOO.

### 4.7. Operational Capability and Overcoming Liability of Outsidership

Strong operational capabilities lead to better products and services, deeper customer insight, and greater buyer appeal. These gains usually increase revenue. Research shows that upgraded OC improves new-product success and overall firm performance (35).

The export manager of Company D stated: “Our A-grade production line and higher quality than Indian rivals have given us a clear edge in new markets.”

Interviewee C noted: “Early investment in technology, modern equipment, and R&D has made exporting easier.”

Interviewee B added: “We quickly spot promising new molecules worldwide. That speed helps us win in low-income markets with flexible rules.”

The managing director of Company H explained: “We adapt products fast, changing brands or packaging to match partner needs. This flexibility beats competitors and smooths market entry.”

The commercial manager of Company E stated: “To offset sanctions, we keep upgrading operations. We improve our marketing mix: better GMP standards for top quality, competitive prices, reliable delivery even when costly, and stronger promotion in formal and informal settings.”

These views suggest that OC builds competitive advantage, strengthens partner trust, and lowers network barriers. We therefore propose the following:

P6: In the internationalization of pharmaceutical companies, operational capability has a positive effect on overcoming LOO.

### 4.8. Integrated Conceptual Framework

**Figure 1.**
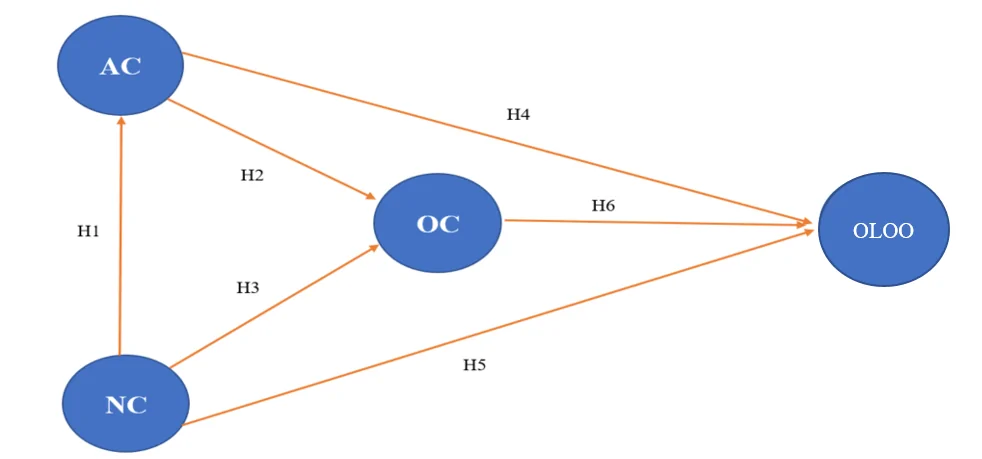
Proposed conceptual model

The conceptual model in this study emerged from the qualitative data analysis and the six propositions outlined above. It draws on organizational capability theories, learning theories, and network theories, all situated within internationalization frameworks such as the revised Uppsala model (10). The model was developed from evidence gathered in Iran’s pharmaceutical industry, a sector under substantial economic and political pressure. Figure 1 shows the resulting framework. The flowchart presents NC -> AC (P1), AC -> OC (P2), NC -> OC (P3), and NC/AC/OC -> overcoming LOO (P4/P5/P6). The nodes represent the three capabilities, and the arrows indicate positive effects.

Figure 1 demonstrates the sequential and synergistic nature of capabilities, emphasizing both direct and indirect paths to addressing sanctions-induced LOO problems.

## 5. Discussion

This study demonstrates how organizational capabilities help pharmaceutical firms in sanctioned emerging economies such as Iran reduce LOO. Using a qualitative multiple-case design, we found that NC and AC act as key dynamic capabilities, while OC provides the operational base. Together, they help firms overcome LOO despite external pressures. These results contribute to international business theory by integrating the Uppsala model, RBV, and dynamic capabilities theory in the context of sanctions.

Overall, the results offer a conceptual synthesis that emphasizes the interdependent nature of the dynamic capabilities, namely NC and AC, and OC. Rather than examining these capabilities in isolation, this synthesis highlights how they jointly create a pathway for dynamic internationalization during crises and enable firms to transform external constraints into strategic opportunities.

From the RBV perspective, the propositions define capabilities as valuable, rare, and inimitable resources that create competitive advantages for companies despite difficult conditions. The classification of P1 to P3 shows how dynamic capabilities, including NC and AC, support OC, thereby transforming resources and increasing efficiency. For example, GMP improvements and more efficient production lines, as described by several managers, translate knowledge gained from international markets into operational capacity. In sanctioned markets, institutional gaps increase isolation; however, NC connects firms to external knowledge and reduces uncertainty, as predicted by dynamic capabilities theory.

Sanctions function primarily as intensifiers of existing liabilities. Nevertheless, this intensification extends the applicability of the Uppsala model by showing how relational limitations transcend cultural or market distance, underscoring the need for alternative networks and resilience strategies. Sanctions emerge as a direct cause of network exclusion, extending the Uppsala model in a novel way. Under sanctions, scarce supplies and logistics disruptions are common (36). Iranian firms use third-country hubs to maintain the movement of goods. Chinese companies facing US restrictions have built self-reliance and new routes, but the present study highlights pharmaceutical regulations and shows how operational upgrades directly counter banking restrictions. This study’s contribution is its treatment of RBV and dynamic capabilities in a tightly regulated field. Capabilities remain dynamic and require ambidexterity through the use of existing resources and the development of new ones (9).

The direct relevance of these capabilities to overcoming LOO (P4-P6) provides a holistic perspective. AC reduces information asymmetry in related markets (37), NC creates social capital, and OC ensures operational legitimacy. These relationships position capabilities as relational instruments rather than merely cost-saving tools. This was illustrated by the manager of Company C, who explained how information from trade fairs and competitors influenced product and country selection decisions. Similarly, the export manager of Company D emphasized the importance of high-quality production lines in competing with Indian companies. Through these mechanisms, organizations become insiders rather than outsiders. They seek regional networks, such as those involving BRICS countries, to avoid dependence on Western connections (38). Third-country representatives and regional partners provide practical applications of networking theories. In highly regulated industries such as pharmaceuticals, legitimacy and compliance standards, including GMP certification, function as signals of reliability. Therefore, operational capability becomes a relational asset that enhances perceived trustworthiness and partially compensates for political exclusion.

Figure 1 links NC, AC, and OC. Previous work has often studied these capabilities separately. The six propositions in this study reveal their joint effects: NC enhances AC, both strengthen OC, and all three reduce LOO. The model provides a dynamic pathway for internationalization under crisis. NC and AC support exploration, whereas OC supports exploitation. Even in harsh settings, firms do not merely adapt; they transform. Strategic capabilities enable them to succeed where others fail.

### 5.1. Practical Implications

Managers can draw direct lessons from this study. They should build NC by forming ties with reliable local agents and regional partners. They should strengthen AC through indirect knowledge sources and staff training. OC should be enhanced through higher production quality and adaptable packaging. This can be achieved through a strategy focused on lower-income countries, where the quality of Iranian products may exceed that of Indian competitors. According to this study, the effective use of internal resources can turn barriers into opportunities for international expansion. These steps help close the gap between theory and day-to-day practice in a restricted economy.

### 5.2. Conclusions

Sanctions do not simply restrict; they also reveal. This study found that Iranian pharmaceutical firms, which face some of the toughest sanctions in the international arena, can turn network exclusion into competitive opportunity through a synergistic triad of organizational capabilities. Based on 10 cases, three major and interrelated paths were identified. NC builds the trust that unlocks knowledge flows; AC converts that knowledge into operational upgrades; and OC translates quality and flexibility into market legitimacy. Together, these capabilities reduce LOO by opening alternative networks, closing information gaps, and signaling reliability to foreign partners. The central insight is not that each capability matters in isolation, but that the capabilities amplify one another. NC feeds AC (P1), AC strengthens OC (P2), NC directly improves OC (P3), and all three collectively reduce LOO (P4-P6). For managers in constrained economies, the practical message is clear: invest in relationships, continuously absorb global knowledge, and allow operational excellence to speak where political access is denied. Adversity, when met with deliberate capability development, becomes a springboard for global integration.

### 5.3. Limitations and Future Research Directions

This study offers useful findings but has limitations. It relies solely on qualitative data. Future work could add quantitative evidence to test the strength of the proposed links and provide a fuller view of how Iranian pharmaceutical firms maintain networks under pressure. Future studies could also include multilevel informants, such as middle managers, operational staff, or external partners, to capture capability enactment at different organizational levels and further validate the proposed relationships.

Several areas remain open for future research. This study did not examine potential mediating factors such as government policies or technology. Differences between generic manufacturers and advanced technology-oriented biotechnology companies were also excluded from the analysis. In addition, the results come from one country and one sector, so applying them elsewhere requires caution.

Comparative studies would be useful. Researchers could study the impact of sanctions by comparing sanctioned countries such as Venezuela with nonsanctioned countries such as India. Future work could also examine internal sector dynamics among generic and innovative organizations, study the long-term effects of sanctions, or investigate nonconventional partners such as blockchain networks and diaspora groups.

## Data Availability

The dataset presented in the study is available on request from the corresponding author after publication. The data are not publicly available due to ethical considerations and confidentiality of interview participants.
